# Awareness, treatment, and control of hypertension is low among adults in Aksum town, northern Ethiopia: A sequential quantitative-qualitative study

**DOI:** 10.1371/journal.pone.0176904

**Published:** 2017-05-10

**Authors:** Teklay Aredehey Gebrihet, Kebede Haile Mesgna, Yosef Sibhatu Gebregiorgis, Alemayehu Bayray Kahsay, Negassie Berhe Weldehaweria, Meresa Gebremedhin Weldu

**Affiliations:** 1 District Health Office, Aksum, Ethiopia; 2 College of Health Sciences, Aksum University, Aksum, Ethiopia; 3 College of Health Sciences, Mekelle University, Mekelle, Ethiopia; University of Alabama at Birmingham, UNITED STATES

## Abstract

**Introduction:**

Hypertension is a major risk factor of cardiovascular diseases which are the leading causes of deaths from chronic non-communicable diseases in Ethiopia. However, little is documented in the issue. Therefore, this study aimed to assess the prevalence, associated factors, awareness, treatment and control of hypertension among adults 18 years old or above in Aksum town, Tigray region, North Ethiopia.

**Methods:**

A sequential quantitative-qualitative study was conducted among adults aged 18 years and above in Aksum town. A multi stage sampling procedure was used to select the study participants for the quantitative study whilst convenience sampling technique was used for the qualitative part. A pre-tested and structured questionnaire was used to collect quantitative data, and an interview guide was used to collect the qualitative data. The logistic regression model was fitted to identify factors independently associated with hypertension using SPSS Version 20. P-values of < 0.05 were considered statistically significant. For the qualitative data, iterative hearing of the discussions verbatim interpretation was followed by categorizing similar ideas into themes and finally triangulated with the quantitative results.

**Results:**

The overall prevalence of hypertension was 16.5% (95% CI: 13.4, 20.0). Awareness, treatment and control of hypertension were 43%, 2.1% and 18.2%, respectively. Being unable to read and write [AOR = 4.73, 95% CI:1.11, 20.23], not consuming fruit [AOR = 4.31, 95% CI:1.74, 10.66], being physically inactive [AOR = 20.11, 95% CI:8.75, 6.20], not knowing physical inactivity is a risk factor of hypertension [AOR = 3.57, 95% CI: 1.69, 7.69] and being overweight/obese [AOR = 9.2, 95% CI:4.54, 18.67] were significantly associated with hypertension. Remarkably, all identified hypertensive cases were linked to the nearby hospital for confirmation of diagnosis, care and follow-up and all of them were found to be hypertensive. This suggests that implementing primary health care approach integrated with the urban health extension package may be effective in the prevention and control of hypertension in poor settings.

**Conclusion and recommendation:**

Prevalence of hypertension among adults was very high, but awareness, treatment and control of hypertension was very low. Being unable to read and write, not consuming fruit, being physically inactive, overweight/obesity and not knowing physical inactivity is a risk factor for hypertension were independently associated with hypertension. Policy makers need to consider integrating prevention and control of hypertension with health extension package. Appropriate information, education and communication strategies should also be designed and implemented to avoid unhealthy lifestyles and promote healthy practices.

## Introduction

Chronic non-communicable diseases (CNCDs) in general and hypertension in particular were originally thought to be associated with affluent societies [1]. Contrary to the common perception of relating CNCDs to the developed world, epidemiologic shift has given rise to an increase in hypertension and other CNCDs in developing countries [1]. Chronic non-communicable diseases account for nearly seven in ten deaths globally and about 40% of these deaths are premature. Sadly, nearly three fourths of all deaths from CNCDs, and an overwhelming majority (>80%) of premature deaths from CNCDs, occur in developing countries[2]. Cardiovascular diseases are the leading causes of deaths from CNCDs which contribute for nearly half of the deaths[2].

Hypertension is the presence of a chronic elevation of systemic arterial pressure above a certain threshold value. Specifically, hypertension is defined as systolic blood pressure ≥140 mmHg or diastolic blood pressure ≥ 90 mmHg or reported use of anti-hypertensive medication in adults aged 18 years and over[3–6]. It is a major cardiovascular problem risk factor and leads to cerebral stroke, congestive heart failure, chronic kidney disease and coronary artery disease[1,7]. Hypertension plays a role in approximately 55% of the global mortality caused by cardiovascular diseases and in 7% of all disability-adjusted life years[8]. The global prevalence of hypertension in adults aged 18 years and over is around 22% in 2014[2].

Africa is one of the regions highly affected by hypertension. More recent study by Adeloye et al. shows that the prevalence of hypertension in Africa is 30.8%[9]. Ethiopia is also one of the most affected countries in the region. According to a recent meta-analysis study in Ethiopia by Kibret et al., the prevalence of hypertension among Ethiopian population is estimated to be 19.6% (95% CI: 13.7%, 25.5%)[10]. This indicates that hypertension is a public health problem in Ethiopia today.

Being preventable with simple and cost effective interventions[8,11], hypertension should be prevented by implementing appropriate preventive measures. Furthermore, people who get hypertensive should be early detected, treated and the raised blood pressure be controlled. Otherwise, low awareness, treatment and control of hypertension lead to life threatening complications, which impacts health and economic gains[11].

Different factors such as socio-demographic characteristics, behavioral factors, bio-medical characteristics, knowledge and attitude could contribute to hypertension[12]. These factors could vary with context. Therefore, research based information regarding prevalence, associated factors, awareness, treatment and control of hypertension are urgently needed for prioritizing, designing and initiating intervention programs aimed at prevention and control of hypertension.

However, previous studies are scarce in the study area and even the limited studies gave emphasis to the prevalence of hypertension and lack comprehensiveness in assessing awareness, treatment and control of hypertension and important variables such as knowledge as a risk factor. Thus, this study is aimed at assessing prevalence, associated factors, awareness, treatment and control of hypertension among adults 18 years old or above in Aksum town, Tigray region, northern Ethiopia to avail the information required for health care providers and policy makers to design and implement appropriate interventions.

## Methods and materials

### Study design, setting and population

A sequential quantitative-qualitative study design was employed from October 1 to December 30/2015 among adults living in Aksum town. Aksum town is located in the northern part of Ethiopia between 14, 1297 (147’46.920”N) latitude and 38, 7158 (3842’56.880”E) longitude at a distance of 1010 km away from Addis Ababa. Administratively the town is divided into four Kebeles (small local administrative units). According to the 2007 report of the Central Statistical Agency of Ethiopia, the total population of Aksum town is 60,766, with 30,991 (51.0%) females and 29,775 (49.0%) males[13]. There is one public hospital and two health centers in the town. Adults aged 18 years or more living in Aksum town were the source population of the study.

### Sample size and sampling technique

The sample size was calculated using OpenEpi. The assumptions for the sample size calculation were: 20.1% prevalence of hypertension among adults from a study by Mengistu et al. in Mekelle town[14], 95% confidence interval, 5% margin of error, design effect of two and 10% non-response rate. The final calculated sample size was 544. A two-stage sampling approach was used to select study participants; where first 2 kebeles were selected randomly from the total 5 kebeles of Aksum town. Then, the total sample size was allocated to each of the randomly selected kebeles based on probability proportional to size allocation. Secondly, 544 households who fulfilled the inclusion criteria were selected by systematic random sampling technique. The first household was selected by lottery method and then the respondents living in every tenth house were recruited. When there is more than one eligible adult in the household, only one individual was selected using the lottery method. Eventually, a total of 521 adults were participating in this study.

Residents who were not at home during the initial contact were re-visited on three other occasions before excluding them from the study. Households were included if: 1) they had non-pregnant adult aged 18 years or older, and 2) the selected adult was a permanent resident (who lived in the area at least for six months) and willing to provide verbal consent. Households were excluded if: 1) no adult resident was at home on more than 3 visits or 2) if the household was vacant.

For the qualitative part, three focus group discussions comprised of 9–12 participants who were not involved in the individual interview were conducted. The focus group discussants were selected using a convenience sampling technique.

### Operational definitions

**Hypertension**: hypertension was defined as systolic blood pressure ≥140 mmHg and/or diastolic blood pressure ≥ 90 mmHg and/or reported use of anti-hypertensive medication[15].

**Controlled hypertension**: blood pressure measurements <140/90 mmHg while on treatment for hypertension were considered as controlled hypertension.

**Body mass index (BMI)**: is calculated as weight in kilograms divided by height in meters squared and interpreted as underweight (BMI<18.5), normal (18.5–24.9), overweight (25.0–29.9) and obese (≥30.0)[15].

**Physical activity level**: physically active if he/she is engaged in moderate-intensity physical activity for at least 150 minutes per week or in vigorous-intensity physical activity for at least 75 minutes per week, or an equivalent combination of moderate and vigorous intensity activity. Whereas, physically inactive if he/she is engaged in moderate -intensity physical activity for < 150 minutes per week, or vigorous intensity physical activity for < 75 minutes per week[16].

**Fruit intake**: good if fruits intake is ≥ 3 days per week and low if fruits intake < 3 days per week[17].

**Vegetable intake**: good if vegetables intake is ≥ 3 days per week and low if vegetables intake of < 3 days per week[17].

**Knowledge**: good if the total knowledge score is above the mean score otherwise low.

### Data collection method and instruments

A structured questionnaire adapted from the World Health Organization instrument for stepwise surveillance (WHO STEPS) of chronic disease risk factors [18] and other relevant literatures [14,19] was used to collect the quantitative data by interview and by taking physical measurements. The questionnaire was contextualized to the local situation (S1 Table). The questionnaire included socio-demographic information, behavioral characteristics (dietary habits, physical activity, alcohol consumption, tobacco use, family history of hypertension, past medical history and drug history), physical measurements and knowledge of hypertension. The quantitative data collection was conducted by eight clinical nurses, who were urban health extension package workers under the supervision of two BSc nurses and the principal investigators. Training and practical demonstrations on interview techniques and anthropometric measurement procedures were given to data collectors for two consecutive days. Prior to data collection, the questionnaire was pre-tested on 5% of the total sample of similar population in one of the non-study kebeles and necessary modifications were made based on the pretest.

The participant’s blood pressure was measured twice in a sitting position using a standard mercury sphygmomanometer with the appropriate cuff size that covers two-thirds of the upper arm after the participant rested for at least five minutes. Participants were inquired whether they had consumed any hot beverage, such as tea or coffee, smoked cigarette or undertaken any vigorous-intensity physical activity 30 minutes before measurement otherwise blood pressure measurement was postponed for 30 minutes. The second measurement was taken five-to-ten minutes after the first measurement. Finally the average of the two blood pressure measurements was computed and used to determine whether participants are hypertensive or not.

Measurement of weight was conducted using a standard beam balance that is used for weight measurement in the medical setup. The scale pointer was checked at zero before taking every measure. Each participant was asked to remove heavy clothes. He/she stood straight and unassisted at the center of the balance platform. Weight measurements were taken to the nearest 0.1 kg.

Height was measured using a wooden height-measuring board with a sliding head bar following standard anthropometric techniques. The participants were asked to remove their shoes, stood erect, and positioned in the Frankfert plane with feet together and knees straight. The heels, buttocks, shoulder blades and the back of the head (occipital) were in touch against the vertical stand of the stadio meter and the values were recorded to the nearest 0.1 cm.

For the qualitative part, a semi-structured open ended interview guide was used as a guide for conducting the focused group discussion. Qualitative data were collected by trained and experienced professionals using tape recording and note taking.

### Data processing and analysis

Data were entered and cleaned using EpiInfo version 6.04, and transferred to SPSS 20 statistical software package for analysis. Descriptive statistics were used to summarize the study variables. Summary measures and proportions of the independent variables were computed against the outcome variable. In this study, the outcome variable was hypertension, which was dichotomized by assigning 1 for those who were hypertensive (systolic blood pressure ≥140 mmHg and/or diastolic blood pressure ≥ 90 mmHg and/or reported use of anti-hypertensive medication) and 0 for those who were not hypertensive (systolic blood pressure <140 mmHg and diastolic blood pressure < 90 mmHg and reported no use of anti-hypertensive medication) during the data collection time. Bivariable analysis was used to see the unadjusted effect of each factor on hypertension. Finally, independent variables with a p-value < 0.05 in the bivariable analysis were fitted into a multivariable logistic regression model to identify their independent effect on hypertension. Adjusted Odds Ratio (AOR) with 95% confidence intervals was estimated to assess the strength of the association, and a p-value < 0.05 was used to declare the statistical significance in the multivariate analysis. Hence, variables with p-value < 0.05 in the multivariate logistic regression analysis were considered as significant and independent determinants of hypertension.

The Hosmer-Lemeshow goodness-of-fit and Omnibus tests of model coefficients tests with enter procedure were used to test for model fitting. The explanatory variables were tested for multicollinearity before entering them into the multivariable model, using the Variance Inflation Factor (VIF) test, the Tolerance test, and values of the standard error.

The qualitative data transcribed and then translated from local language, Tigrigna to English in verbatim manner. After the interviews, listened to many times, the transcripts were reduced, coded and categorized into themes, and finally triangulated with the quantitative results.

### Ethical consideration

Ethical clearance was approved by the Institutional Research Review Board of College of Health Sciences and Referral Hospital, Aksum University. Permission letter was also obtained from Aksum district health office. Moreover, all study participants were informed about the purpose of the study and verbal consent was obtained from each study participant before data collection. Confidentiality was ensured by using a questionnaire identification number in which was immediately detached and filed separately in a confidential manner. Subjects with high blood pressure (hypertension) were linked to health facilities for care and follow-up.

## Results

### Socio-demographic characteristics of study participants

A total of 521 respondents was included in this study, making the response rate of 96%. The mean (±SD) age of respondents was 36.44 (± 12.4). Nearly half of the respondents (48.6%) were in the age group of 30–49 years. More than half (61%) were females. Of the total respondents, 516 (99%) were Tigray in ethnicity, 421 (80.8%) were Orthodox in religion and 350 (67.2%) were married. Majority 282 (54.2%) attended at most primary level education or not at all. About one fourth (25.5%) were house wives followed by merchants (24.0%) and employed (19.5%). The median monthly family income was 1,000.00 ETB, and 217 (41.7%) of respondents were in the family income category of <1000.00 ETB per month (Table 1).

**Table 1:** Socio-demographic characteristics of the respondents in Aksum town, Tigray, northern Ethiopia, 2015 (n = 521).

Characteristics		Frequency	%
**Sex**	Male	203	39.0
	Female	318	61.0
**Age group**	18–29	178	34.2
	30–49	253	48.6
	≥50	90	17.3
**Educational status**	Unable to read and write	90	17.3
	Primary	192	36.9
	Secondary	164	31.5
	Above secondary	75	14.4
**Ethnicity**	Tigray	516	99.0
	Other*	5	1.0
**Religio**n	Orthodox	421	80.8
	Muslim	99	19.0
	Catholic	1	0.2
**Marital status**	Married	350	67.2
	Single	89	17.1
	Divorced	50	9.6
	Widowed	32	6.1
**Occupational statu**s	House wife	135	25.9
	Merchant	125	24.0
	Employed**	102	19.5
	Daily laborer	77	14.8
	Student	49	9.4
	No job	21	4.0
	Farmer	12	2.3
**Monthly family income**	≤1000 ETB***	217	41.7
	1001–2000 ETB	207	39.
	2001–3000 ETB	37	7.1
	>3000 ETB	60	11.5

* = Amhara, Oromo and Gurage

** = indicates employment in both governmental and non-governmental organizations

*** = Ethiopian Birr

### Behavioral, dietary and bio-medical related characteristics

Of the total respondents, seven (1.3%) were current smokers and 154 (29.6%) were current alcohol users. Four hundred twenty eight (82.1%) of the total respondents reported that they eat fruits, but only 89 (17.1%) eats a fruit at least three days per week. Almost all of the respondents 500 (96%) reported that they eat vegetable in their servings, but only 138 (26.5%) eats three days or more per week. Majority 442 (84.8%) of the respondents reported that they use saturated fat and oil in their diet. Of the total respondents included in this study, 341 (65.5%) were physically active. Regarding to body mass index, 82 (15.7%) of respondents were obese/overweight (BMI ≥25 kg/m^2^). In addition, 41 (7.9%) of the respondents had a family history of hypertension (Table 2).

**Table 2:** Behavioral, dietary and bio-medical related characteristics of the respondents in Aksum town, Tigray, northern Ethiopia, 2015 (n = 521).

Characteristic		Frequency	%
**Smoking**	Never	505	97.0
	Currently	7	1.3
	Previous	9	1.7
**Alcohol use**	Never	312	59.9
	Currently	154	29.6
	Previous	55	10.5
**Frequency of drinking alcohol/week**	<3 days	124	23.8
	≥3 days	30	5.8
**Consume fru**it	Yes	428	82.1
	No	93	17.9
**Fruit consumption/week**	<3 days	339	65.0
	≥3 days	89	17.1
**Consume vegetable**	Yes	500	96.0
	No	21	4.0
**Vegetable consumption/week**	<3 days	362	69.5
	≥3 days	138	26.5
**Type of fat/oil most often used**	Unsaturated	79	15.2
	Saturated	442	84.8
**Physical activity**	Active	341	65.5
	Inactive	180	34.5
**BMI (kg/m**^**2**^)	Under weight	42	8.1
	Normal	397	76.2
	Overweight/obese	82	15.7
**Family history of hypertension**	Yes	41	7.9
	No	480	92.1
**Family member with hypertension**	Parents	21	4.0
	Siblings	9	1.8

BMI = Body Mass Index

### Knowledge of respondents on hypertension

The most common risk factors of hypertension identified were high salt intake 318 (61%), high fat intake 270 (51.8%), obesity 253 (49.1%), and physical inactivity 189 (36.3). About 480 (92.1%) reported that hypertension is preventable and the commonly reported prevention methods were decreasing salt intake 371 (71.2%), physical activity 318 (60%) and decreasing fat intake 257 (49.3%). Generally, more than half (51.65%) of the respondents had low knowledge of risk factors and prevention methods of hypertension (Table 3).

**Table 3:** Knowledge on risk factors and prevention methods of hypertension among adults in Aksum town, Tigray, northern Ethiopia, 2015 (n = 521).

Characteristic		Frequency	%
**Ever heard about hypertension**	Yes	491	94.2
	No	30	5.8
Knowledge on risk factors of hypertension*	High salt intake	318	61.0
	High fat intake	270	51.8
	Obesity	256	49.1
	Physical inactivity	189	36.3
	Alcoholism	157	30.1
	Stress	106	20.3
	Advancing in age	84	16.1
	Smoking	40	7.7
	Coffee	30	5.8
	Diabetes Mellitus	8	0.4
**Knows that hypertension is preventable**	Yes	480	92.1
	No	41	7.9
Knowledge on prevention methods of hypertension*	Decreasing salt intake	371	71.2
	Doing physical activity	318	61.0
	Decreasing fat intake	257	49.3
	Not using alcohol	218	41.8
	Not smoking	39	7.5
	Others**	18	3.45
**Composite knowledge score**	Low	269	51.6
	High	252	48.4

* = the percentages do not add up to 100% due to multiple responses

** = reducing sugar intake, coffee and stress

### Qualitative findings concerning knowledge on hypertension

The socio-demographic characteristic of the discussants was, 14 (46.7%) were women and the rest 16 (53.3%) were males. All were older than 18 years in age and residents of Aksum town. Two main themes, namely risk factors of hypertension and prevention methods of hypertension were identified in the thematic framework analysis. Accordingly the findings are presented as follows.

### Knowledge on risk factors of hypertension

The focus group discussion identified lack of knowledge on risk factors of hypertension. Concerning risk factors of hypertension, feeding habit was mentioned by most of the discussants. However, they failed to name risky feeding habit. For instance, a 42 years old woman claimed:

“… Eating too much food of any type is the most common risk factor which exposes people to hypertension …………..”

Lack of knowledge on risk factors of hypertension was also substantiated by another 50 years old man who stated:

“… Generally, I believe that it is chance or God’s will which exposes people to hypertension not any other factor. But sometimes, eating too much food, especially too sweet food such as honey, consuming local crop, red ‘teff’ and red root increased the risk of getting hypertension….”

Some discussants that had better awareness regarding the risk factors of hypertension were able to mention fat, salt and coffee as risk factors. However, still they failed to mention types of food with high fat content. For instance, a 30 years old woman claimed:

*“.…High salt intake exposes people with hypertension. In addition, high fat intake increases the risk of getting hypertension. ……………..High fat containing foods are oil of any type and any sweet foods such as honey”*.

### Knowledge on prevention methods of hypertension

Similar to that of risk factors for hypertension, the focus group discussion identified lack of knowledge on preventive measures of hypertension. Most of the discussants revealed that hypertension is preventable. When they are asked to mention the preventive measures, most of them declared that it can be prevented by reducing salt and coffee. However, all of them reported that they are not practicing it. In addition, avoiding high fat intake was stated by most of the discussants. However, they failed to name clearly the recommended dietary practice or what to avoid and what to consume. For instance, a man of 37 years old revealed: *“*… *I believe that hypertension is preventable by improving dietary practice*, *particularly by avoiding high fat intake just by avoiding consuming oil and sweet foods*. *In addition*, *it is important to have medical checkup ……”* On the other hand, most of the discussants failed to mention exercise, reducing stress, avoiding alcohol, avoiding smoking and controlling body weight as preventive measures of hypertension.

### Prevalence, awareness, treatment, and control of hypertension

The overall prevalence of hypertension was 16.5% [95% CI: 13.4, 20.0]. Among all hypertensive individuals identified, more than half (57%) were not aware of their hypertension status. More than two third, 59 (68.6%) of the respondents have had their blood pressure measured at least once before by a doctor or other health professional, but only 37 (43%) have been told before that they are hypertensive. Moreover, only 11 (12.8%) were taking drugs (medication) for hypertension and the overall control rate of hypertension was only 2 (18.2%) (Table 4).

**Table 4:** Prevalence, awareness, treatment, and control of hypertension among adults in Aksum town, Tigray, northern Ethiopia, 2015 (n = 521).

Characteristics	Frequency	%
Currently hypertensive (n = 521)	86	16.5
Ever measured blood pressure (n = 86)	59	68.6
Ever been told by a doctor or other health professional that they have hypertension (n = 86)	37	43.0
Have taken anti-hypertensive medication (n = 86)	11	12.8
Hypertension controlled (n = 11)	2	18.2

### Factors associated with hypertension

This study examined a number of independent variables with the outcome variable (hypertension) in both bivariable and multivariable analyses levels. Socio-demographic characteristics (sex, age group, educational status, ethnicity, religion, marital status, occupational status and monthly family income); behavioral, dietary and bio-medical related characteristics (smoking, alcohol use in life and frequency per week, consuming fruit and consumption per week, consuming vegetable and consumption per week, type of fat/oil most often used, physical activity, Body Mass Index (kg/m^2^), family history of hypertension and member with hypertension) and knowledge on risk factors and prevention methods of hypertension (ever heard about hypertension, knowledge on risk factors and prevention methods of hypertension, knows that hypertension is preventable and composite knowledge score) were analyzed to identify candidate variables for the final model. Among these examined independent variables: age group, low level of education, physical inactivity, not consuming fruit and vegetable, family history of hypertension, overweight/obesity and not knowing physical inactivity as a risk factor for hypertension were significantly associated with hypertension in the bivariable regression analysis.

All identified candidate variables such as age group, low level of education, physical inactivity, not consuming fruit and vegetable, family history of hypertension, overweight/obesity and not knowing physical inactivity as a risk factor for hypertension were also assessed in the final model and multivariable logistic regression analysis revealed that educational status was associated with hypertension. The likelihood of being hypertensive was about five times more likely among respondents who were unable to read and write [AOR = 4.73, 95% CI; 1.11, 20.23] compared to those with above secondary education. Physically inactive respondents [AOR = 20.11, 95% CI; 8.75, 46.20] were about 20 times more likely to develop hypertension than those who were physically active. Knowledge on physical inactivity as a risk factor was also associated with hypertension; respondents who didn’t know physical inactivity as a risk factor of hypertension [AOR = 3.57, 95% CI; 1.69, 7.69] were more likely to be hypertensive than their counterparts. Moreover, overweight/obese (BMI≥25 kg/m^2^) respondents [AOR = 9.2, 95% CI; 4.54, 18.67] were at more than nine times at increased risk of being hypertensive compared to those with normal BMI. Likewise, respondents who did not consume fruit [AOR = 4.31, 95% CI; 1.74, 10.66] were more likely to be hypertensive (Table 5).

**Table 5:** Bivariable and multivariable logistic regression analyses of factors associated with hypertension among adults in Aksum town, Tigray, northern Ethiopia, 2015 (n = 521).

Variables		Hypertensive		COR (95% CI)	AOR (95% CI)
		Yes n (%)	No n (%)		
**Age group (years)**	18–29	11 (6.2)	167 (93.8)	1	1
	30–49	45 (17.8)	208 (82.2)	3.29 (1.65, 6.55)	0.50 (0.17, 1.48)
	≥50	30 (33.3)	60 (66.7)	7.59 (3.58, 16.09)	0.77 (0.34, 1.77)
**Educational status**	Unable to read and write	26 (28.9)	64 (71.1)	3.40 (1.43, 8.07)	4.73 (1.11, 20.23)*
	Primary	39 (20.3)	153 (79.7)	2.16 (0.95, 4.81)	3.13 (0.87, 11.23)
	Secondary	13 (7.9)	151 (92.1)	0.72 (0.29, 1.82)	1.28 (0.33, 4.93)
	Above secondary	8 (10.7)	67 (89.3)	1	1
**Fruit consumption**	Yes	56 (13.1)	372 (86.9)	1	1
	No	30 (32.3)	63 (67.7)	3.163 (1.89, 5.31)	4.30 (1.74, 10.66)*
**Vegetable use**	Yes	79 (15.8)	421(84.2)	1	1
	No	7 (33.3)	14 (66.7)	2.66 (1.04, 6.81)	1.25 (0.29, 5.43)
**Physical activity**	Active	16 (4.7)	325 (95.3)	1	1
	Inactive	70 (38.9)	110 (61.1)	12.93 (7.20, 23.2)	20.11 (8.75, 46.20)*
**Family history of hypertension**	Yes	16 (39)	25 (61)	3.75 (1.90, 7.37)	0.41(0.15, 1.15)
	No	70 (14.6)	410 (85.4)	1	1
**Knowledge on physical inactivity as a risk factor of hypertension**	Yes	12 (6.3)	177 (93.7)	1	1
	No	74 (22.2)	258 (77.7)	4.23 (2.23, 8.00)	3.57 (1.69, 7.69) *
**Knowledge on stress as a risk factor of hypertension**	Yes	25 (23.6)	81 (76.4)	1	1
	No	61 (14.7)	354 (85.3)	0.56 (0.33, 0.94)	0.57 (0.26, 1.25)
**BMI (kg/m**^**2**^**)**	Under weight	2 (4.8)	40 (95.2)	0.55 (0.13, 2.39)	0.30 (0.05, 1.69)
	Overweight/obesity	51 (62.2)	31 (37.8)	18.15 (10.25, 32.12)	9.21 (4.54, 18.67) *
	Normal	33 (8.3)	364 (91.7)	1	1

AOR = Adjusted Odds Ratio

COR = Crude Odds Ratio

CI = Confidence Interval

BMI = Body Mass Index

* = statistically significant at p value of < 0.05

## Discussion

This community based cross-sectional study conducted in Aksum town assessed the prevalence and factors associated with hypertension. In this study, the prevalence of hypertension was 16.5% (95% CI: 13.4, 20.0). This finding is consistent with a recently conducted meta-analysis study on the prevalence of hypertension (19.6%, 95% CI: 13.7, 25.5) in Ethiopia[10]. However, the finding of this current study is higher than the finding of similar studies in Ethiopia; in southwest Ethiopia (13.2%), in northwest Ethiopia (13.3%), and in southern Ethiopia (10.1%)[20–22]. The observed discrepancy could be due to difference in study time, which suggests that the prevalence of hypertension is increasing over time in Ethiopia.

Conversely, the finding of the current study is lower than the findings of other similar studies in Ethiopia: Gondar (28.3%), Durame (22.4%) and Addis Ababa (30%)[19,23,24]. This could be due to the fact that age is positively related with hypertension and all of these previous studies recruited older participants (≥34 years in Gondar, >31 in Durame and ≥25 years in Addis Ababa) compared to the current study which recruited ≥18 years adults. In the current study a significant proportion (34%) of the respondents were between 18 and 29 years. Similarly, the finding of the current study is lower than the findings of similar studies elsewhere; a recently conducted systematic analysis in Africa (30.8%)[9], and in Europe (28.0%–44.0%)[25]. The discrepancy could be due to the difference in lifestyle.

Furthermore, common modifiable risk factors of hypertension such as saturated fats/oil use (84.8%), low fruit consumption (82.9%), low vegetable use (73.5%), physical inactivity (34.5%), alcohol use (29.6%) and overweight/obesity (15.7%) were very prevalent in the current study. On top of that, more than half of the respondents had low knowledge on the risk factors and prevention methods of hypertension. This was supported by the qualitative findings. This suggests that there may be a large number of people who are at risk of hypertension and other common cardiovascular diseases in the community at large.

A remarkable finding of the current study is that a large proportion (57%) of those with hypertension did not know their hypertension status before the study. This is in line with a study conducted in Southwest Ethiopia, where nearly 55% of hypertensive patients were newly screened at the time of the study[26]. Moreover, more than three fourth of those who were already aware of their hypertension status were not on treatment which is consistent with the finding of a similar study in Southwest Ethiopia[20]. This was also supported by the qualitative findings in which discussants stated that people diagnosed to have hypertension do not prefer to start medication because it is believed that taking antihypertensive drugs is harmful. Furthermore, the overall hypertension control rate was very low even among those who were on treatment, which is in agreement with the finding of a similar study in Ethiopia[20]. These findings indicate that there may be a large number of people who have hypertension in the community, but are not aware of it. When this disease goes undiagnosed they can cause further complications. So, there is an urgent need to find a means to address this gap.

Another striking finding of this study is all identified hypertensive respondents were linked to the nearby hospital for confirmation of diagnosis, care and follow-up and all of them were found to be hypertensive. Hence, this implies that it is possible to screen and identify people with hypertension, common modifiable risk factors of hypertension and other common cardiovascular diseases using Health Extension Program (HEP) that has been implemented at grass root level in the Ethiopian community for the maternal and communicable disease interventions. This suggests that implementing primary health care approach integrated with the urban health extension package may be effective in the prevention and control of the silent epidemic of common chronic non-communicable diseases in poor settings like Ethiopia.

Concerning the factors associated with hypertension, the results of the current study revealed that education was inversely associated with hypertension. The likelihood of being hypertensive was significantly higher among respondents who are unable to read and write compared to those with above secondary education. The odds of being hypertensive decreased as education level increased. This finding is in line with other community based studies conducted in Ethiopia, USA, China, and Italy[27–30]. This could be due to the fact that higher level of education results in better awareness of the risk factors and preventive measures of hypertension[31–34]; leads to healthy lifestyle and lower risk of getting hypertensive.

As expected, physical activity was significantly associated with hypertension; physically inactive respondents were more likely to develop hypertension than their counterparts. This finding is also in agreement with studies conducted in Ethiopia, India and USA[19,23,35,36]. As evidence has proven that physical activity influences a number of physiological functions such as improving insulin sensitivity, decreasing blood glucose and blood pressure levels, weight reduction, cholesterol reduction, increasing muscle tone, improving circulation, stress relief and well-being feelings[37].

Knowing physical inactivity as a risk factor of hypertension was also significantly associated with hypertension; respondents who didn’t know physical inactivity is a risk factor of hypertension were more likely to be hypertensive than their counterparts. This is supported by a similar study from Karachi, Pakistan which showed that knowing changing lifestyle improves blood pressure results in controlled hypertension compared to their counterparts[38]. In addition, the prevalence of hypertension has been slightly decreased with higher knowledge on hypertension in a study conducted in rural areas of China[34]. On top of that, the likelihood of eating salty pickled vegetable at least once in the last week was significantly lower among those who knew eating less salt usually makes blood pressure go down[34]. This points out that not knowing physical inactivity as a risk factor for hypertension can predispose individuals to experience a sedentary lifestyle which eventually leads to hypertension.

Among the dietary related characteristics assessed in this study, fruit consumption was statistically associated with hypertension; respondents who didn’t consume fruit were more likely to be hypertensive than their counterparts. This finding is in line with other studies elsewhere[39–41]. This could be partly explained by fruit intake reduces risk of hypertension through weight reduction as evidenced by the finding of study done elsewhere[42].

Furthermore, BMI was independently associated with hypertension; over-weighted/obese respondents were more likely to be hypertensive compared to their normal counterparts. This finding is in line with study across three populations in Africa and Asia; Ethiopia, Vietnam and Indonesia, India and other studies from Sub-Saharan Africa countries; Ethiopia, Uganda and Kenya[5,15,19,20,22,24,35,43–46]. The observed relationship could be due to the difference in dietary habits and physical inactivity of study participants.

Although a family history of hypertension, history of smoking, and alcohol consumption status were independently associated with hypertension in other studies in Ethiopia, Uganda, and India[19,23,46–49], no such associations were found in this study. This might be due to the low prevalence of these risk factors in the current study respondents.

This study has its own drawbacks. The study suffers from the usual limitation of cross sectional study. It did not assess the contribution of blood glucose and blood lipids (triglycerides, high density lipoprotein (HDL) cholesterol and total cholesterol). In addition, the study was limited to urban population only which may limit its generalizability. Furthermore, this study is not free from recall and social desirability biases.

## Conclusion

The prevalence of hypertension was very high. In addition, awareness, treatment and control rate of hypertension was very low. Risk factors for hypertension were highly prevalent. The findings of this study revealed that inability to read and write, low fruit eating habit, physical inactivity, overweight/obesity and not knowing physical inactivity as a risk factor for hypertension were significantly associated with hypertension. Policy makers need to consider integrating prevention and control of hypertension with health extension package. Appropriate information, education and communication strategies should be designed and implemented to avoid unhealthy lifestyles and promote healthy practices.

## Supporting information

S1 Table Hypertension English and Tigrigna version tool. (DOCX)
