# Supplementary material for: Awareness, treatment, and control of hypertension is low among adults in Aksum town, northern Ethiopia: A sequential quantitative-qualitative study
Source: PLoS One. 2017 May 10;12(5):e0176904. doi: 10.1371/journal.pone.0176904 (PMC5425176; doi:10.1371/journal.pone.0176904)
Supplement: S1 Table — (DOCX) [file pone.0176904.s001.docx]

## Annex 2: English version questionnaire

Date”_______/_____/2015

**Informed verbal consent Form**

Aksum University, College of Health Sciences and Referral Hospital, Department of Public Health

Questionnaire on the prevalence of hypertension and associated factors among adults in Aksum town, Tigray, northern Ethiopia: community based cross-sectional study design My name is **___________.** This study is conducting on the prevalence of hypertension and associated factors among adults in Aksum town, Tigray, northern Ethiopia by Aksum University College of Health Sciences and Referral Hospital in collaboration with Woreda Health Office of Aksum town.. I will ask you some not much difficult questions and measuring your blood pressure. Whether you are willing or not to participate in this study; there is no negative implication on your health care services provision. You have right to withdraw at any time in the interviewing and taking measurements processes when you feel discomfort in participating in this study. The study will use only code numbers, protect you from the risks of breach of confidentiality and invasion of privacy; however, this study will be realized and generated accurate findings on the prevalence of hypertension and associated factors among adults in Aksum town, Tigray, northern Ethiopia and public health and clinical implication in awareness, treatment and control rate of hypertension. If your blood pressure identifies you as having hypertension; you will be convinced and linked to nearby hospital for treatment and follow up.

We value your input to make this study a successful one. It will take only 20 minutes.

Do you agree to participate in this study?

Yes: No: if yes, continue to the next

If No, thank you for your time.

Interviewer’s Name: _______________________Signature: ________________

| Questionnaire Code Number |
| --- |
|  |

**Part I: Socio-demographic characteristics related questions**

| S.No | Questions | Responses | Skip Pattern | Codes |
| --- | --- | --- | --- | --- |
| 101 | Sex of study participant | 1. Male 2. Female |  | **[ ]** |
| 102 | What is your age? (year) | __________ |  | **[ ]** |
| 103 | What is the highest level of education you have completed? | 1. Unable to read and write 2. Primary school (1-8) completed   3. High school (9-12) completed  4. Collage /university/ completed  5. Post graduate degree |  | **[ ]** |
| 104 | What is your Ethnicity? | 1. Tigray  2. Other ( specify) ___________ |  | **[ ]** |
| 105 | What is your Religion? | 1. Orthodox 2. Muslim 3. Protestant 4. Catholic 5. Other (Specify)________ |  | **[ ]** |
| 106 | What is your marital status? | 1. Single 2. Married 3. Divorced 4. Widowed |  | **[ ]** |
| 107 | What is your occupational status? | 1. Government employee 2. Non-government employee 3. Merchant 4. Student 5. Daily laborer 6. Other( specify) ___________ |  | **[ ]** |
| 108 | How many people older than 18 years, including yourself, live in your household? | ___________(in number) |  | **[ ]** |
| 109 | What is your monthly income in birr? | ___________ |  | **[ ]** |

**II. Behavioral related Questions**

| S.No | Questions | Responses | | Skip Pattern | Codes |
| --- | --- | --- | --- | --- | --- |
| **Tobacco use** | | | | | |
| 110 | Have you ever smoke any tobacco products, such as cigarettes? | 1. Yes 2. No | If No skip to No 116 | | **[ ]** |
| 111 | Do you currently smoke any tobacco products, such as cigarettes? | 1. Yes 2. No | If No skip to No 115 | | **[ ]** |
| 112 | Do you smoke daily? | 1. Yes 2. No |  | | **[ ]** |
| 113 | Do you remember for how many years you were smoking? | ____________(years)  Don’t remember (77) |  | | **[ ]** |
| 114 | On average, how many cigarettes do you smoke each day? | ______(number of cigarettes) |  | | **[ ]** |
| 115 | How long ago did you stop smoking daily?  (Record only one, not all) | ________(years)  ________ (months)  ________(weeks)  Don’t remember (77) |  | | **[ ]** |
| **Alcohol Consumption** | | | | | |
| 116 | Have you ever consumed alcohol (such as beer, wine,) in your life? | 1. Yes 2. No | If No skip to No 122 | | **[ ]** |
| 117 | Have you consumed any alcohol within the past 12 months? | 1. Yes 2. No |  | | **[ ]** |
| 118 | Do you currently drink alcohol? | 1. Yes 2. No | If No skip to No 122 | | **[ ]** |
| 119 | During the past 12 months, how frequently have you had at least one standard alcoholic drink? | 1. Daily 2. 5-6 days per week 3. 3-4 days per week 4. 1-2 days per week 5. 1-3 days per month 6. Less than once a month 7. Other (specify) __________ |  | | **[ ]** |
| 120 | During the past 30 days, how many standard drinks on average did you have during one drinking occasion? | Number ________  Don't know …77 |  | | **[ ]** |
| 121 | On average, how many standard drinks per week do you drink? | Number of drinks ___ ___  Don't know ….77 |  | | **[ ]** |
| **Diet** | | | | | |
| 122 | Do you eat fruits? | 1. Yes 2. No | If No skip to No 125 | | **[ ]** |
| 123 | On average how many days do you eat fruits in a week? | _______(days) |  | | **[ ]** |
| 124 | How many servings of fruit do you eat on one of those days? | _______(serving number)  Don't Know (77) |  | | **[ ]** |
| 125 | Do you eat vegetables? | 1. Yes 2. No | If No skip to No 129 | | **[ ]** |
| 126 | In a typical week, on how many days do you eat vegetables? | ________(days) Don't Know (77) |  | | **[ ]** |
| 127 | How many servings of vegetables do you eat on one of those days? | _______(serving number)  Don't Know (77) |  | | **[ ]** |
| 128 | When you prepared meal in your house hold what type of oil or fat is most often used?  (Select only one) | 1. Vegetable oil 2. Butter 3. Margarine 4. Don’t use any 5. Don’t know (77) |  | | **[ ]** |
| **Physical Activity** | | | | | |
| 129 | Have you involved vigorous intensive activity that causes large increases in breathing or heart rate for at least 10 minutes continuously? | 1. Yes 2. No | If No skip to No 132 | | **[ ]** |
| 130 | On average how many days do you do vigorous intensive activities as part of your work in a week? | ________(days) |  | | **[ ]** |
| 131 | How much time do you spend doing vigorous-intensive activities at work on a typical day? | ______(hours)  ______(minutes) |  | | **[ ]** |
| 132 | Does your work involve moderate-intensive activity that causes small increases in breathing or heart rate such as brisk walking for at least 10 minutes continuously? | 1. Yes 2. No | If No skip to No 135 | | **[ ]** |
| 133 | On average how many days do you do moderate intensive activities as part of your work in a typical week? | ________(days) |  | | **[ ]** |
| 134 | How much time do you spend doing moderate-intensity activities at work on a typical day? | ______(hours)  ______(minutes) |  | | **[ ]** |

**III. Bio-medical related questions**

| S.No | Questions | Responses | Skip Pattern | Codes |
| --- | --- | --- | --- | --- |
| 135 | Have you ever measured your blood pressure by doctor or other health professional? | 1. Yes 2. No | If No skip to 139 | **[ ]** |
| 136 | Have you ever been told by a doctor or other health worker that you have hypertension? | 1. Yes 2. No |  | **[ ]** |
| 137 | Have you been told in the past 12 months? | 1. Yes 2. No |  | **[ ]** |
| 138 | In the past two weeks, have you taken any drugs (medication) for hypertension prescribed by a doctor or other health worker? | 1. Yes 2. No |  | **[ ]** |
| 139 | In your family is there any one with hypertension? | 1. Yes 2. No | If No skip to 141 | **[ ]** |
| 140 | Who is with hypertension in your family? | 1. Parents 2. Sister(s) 3. Brother(s) |  | **[ ]** |

**IV. Knowledge on hypertension questions**

| S.No | Questions | Responses | Skip Pattern | Codes |
| --- | --- | --- | --- | --- |
| 141 | Have you ever heard about hypertension? | 1. Yes 2. No | If No skip to 146 | **[ ]** |
| 142 | What ceases hypertension | 1. Increase volume of blood in our body 2. Hereditary 3. Other (specify)_________ |  | **[ ]** |
| 143 | What are the main risk factors for hypertension | 1. Over weight /obesity 2. High fat intake 3. High salt intake 4. Smoking 5. Alcoholism 6. Low physical activity 7. Higher age 8. Other (specify)_________ |  | **[ ]** |
| 144 | Can hypertension be prevented? | 1. Yes 2. No |  | **[ ]** |
| 145 | How can hypertension be prevented? | 1. Physical exercise 2. Not drinking alcoholic drinks 3. Not smoking 4. Use low fat content foods 5. Use low salt content foods 6. Other (specify)___________ |  | **[ ]** |

**V. Physical body measurements**

| S.No | Questions | Response | Skip pattern |
| --- | --- | --- | --- |
| 146 | **Blood pressure** | | |
|  | Blood Pressure: reading 1 | Systolic(mmHg) _________  Diastolic(mmHg)_________ |  |
|  | Blood Pressure: reading 2 | Systolic(mmHg) _________  Diastolic(mmHg)_________ |  |
|  | Blood Pressure: reading 3 | Systolic(mmHg) _________  Diastolic(mmHg)_________ |  |
| **Height and Weight** | | | |
| 147 | Height (cm) | _____________ |  |
| 148 | Weight (kg) | _____________ |  |
| 149 | Calculated BMI (Kg/m^2^) | _____________ |  |

**Thank you for your time**

##

## Tigrigna Version Questionnaire

## ትግርኛ ሰነድ ሓበሬታ መፅናዕቲ

ኣክሱም ዩኒቨርሲቲ ኮሌጅ ጥዕና ሳይንስ ሪፈራል ሆስፒታል ክፍሊ ትምህርቲ ጥዕና ሕብረተሰብ መፅናዕቲ ፈተሸ ዝረገሐን ጠንቂታትን ሕማም ፀቅጢ ደም ኣብ ከተማ ኣክሱም ትግራይ ስሜናዊ ኢትዮጵያ

**ጥዕና ይሃበለይ!**

ኣክሱም ዪኒቨርሲቲ ኮሌጅ ጥዕና ሳይንስ ሪፈራል ሆስፒታል ክፍሊ ትምህርቲ ጥዕና ሕ/ሰብ

መሕትት ቅጥዒ ኣብ ሕብረተሰብ ውሽጢ ተርእዮታት ሕማም ፀቅጢ ደም ስፍሓቱን ተዛመድቱን እቲ ኩነት ብሕ/ሰብ ደረጃ ኣብ ከተማ አክሱም፣ ትግራይ፣ ሰሜን ኢትዮጵያ እንታይ ከም ዝመስል ኣብ ምፅናዕ ይርከብ**::**

ሽመይ___________ይበሃል፤ ብምትሕብባር ኣክሱም ዩኒቨርሲቲን ቤት ፅሕፈት ጥዕና ከተማ ኣክሱም መፅናዕቲ ንምክያድ ቃለ-መሕተት ንነባሪ ከተማ አክሱም ደረጃ ብሕ/ሰብ ዝርገሐ ፀቕጢ ደምን መንቀልታቱን ኣብ መፅናዕ ንርከብ:: ከበድቲ ዘይኮኑ ዝተወሰኑ ሕቶታት ክሓኩም/ክን እየ፤ ከምእውን ዓቐን ፀቕጢ ደምኩም/ክን ንምፍላጥ ብመሳሪሒ ክልከዐኩም/ክን እየ:: ኣብ’ዚ መፅናዕቲ ብምስታፍኩም/ክን ወይ ብዘይምስታፍኩም/ክን ኣብ ቀፃላይ ትረኽብዎ/ኦ ግልጋሎት ጥዕና ምንም ዓይነት ኣሉታዊ ፅልዋ ኣይህልዎን፤ እቲ ሕቶን መልስን ወይ ከዓ እቲ መሳሪሒ መዕቀኒ ፀቕጢ ደም ዘይደለኹምዎ/ኽንኦ እንተኮይኑ ኣብ ዝኾነ እዋን ክተቋርፅዎ/ኦ ትኽእሉ/ላ እኹም/ኽን:: ሽምኩም/ክን እውን ምግላፅ ይኹን ምሳኹም/ኽን ጥቆማታት ዘይተሓሓዙ መለለይ ቁፅሪ መሕተቲ ቅጥዒ ጥራሕ ኢና ንጥቀም፤ ኮይኑ ግና ሓቀኛ ዝኾነ መልስን ቅኑዕ ትሕብብርኩም/ክን ነዚ እንገብሮ መፅናዕቲ ብሉፅ ዝኾነ እታዎት ስለ ዝህልዎ ኣብቲ ሕ/ሰብና ዝረኣዩ ሕማም ፀቅጢ ደም ስፍሓቱን ተዛመድቱን እቲ ኩነት ብሕ/ሰብ ደረጃ ንምፍታሕ ዝዓዘዘ ጥቅሚ ኣለዎ። ከም ኣጋጣሚ ደኩም/ክን ልዑል ፀቕጢ እንተመኡ ማለት ደም በዝሒ እንተሃሊዩ ናብ ቀረባ ሆስፒታል ንሕክምና ክንልእኾም/ኽን ኢና::

ንእትገብሩልና/ራልና ዘይተሓለለ ምትሕብባር ካብ ልቢ ነመስግን፣ እዚ መሕተት ኣስታት 20 ደቂቃ ክወስድ ይኽእል እዩ።

1. ተሓታታይ ተሰማሚዖም/ዐን ቀፅል
2. ተሓታታይ ኣይተሰማመዑን/ዓን የቐንየለይ!

ናይ ሓታታይ ሽምን ፊርማን________________________________________

| Ýl-መሕትት mFlY ቁፅሪ |
| --- |
|  |

መዘኻኸሪ **-** ካብቶም ዝተውሃቡ መማረፅታት ብምክባብ ሕረ/ዪ፤ ካሊእ ሓሳብ እንተሃልዩ ኣብቲ ክፍቲ ቦታ ይፅሓፉ/ፋ

**ሀ. ማሕበራዊን ኢኮኖሚያዊን መስተጋብራት ዝምልከቱ ሕቶታት**

| ተ.ቁ | ሕቶታት | መልሲ | ናብ ዝቕፅል ሕለፉ/ፋ | መፍለጢ ቁፅሪ |
| --- | --- | --- | --- | --- |
| 101 | ፆታ ተሳታፊ እቲ መፅናዕቲ | 1. 1. ተባዕታይ 2. 2. ኣንስታይ |  | [ ] |
| 102 | ዕድሜኹም/ኽን ክንደይ እዩ? (ብዓመት) | ______ |  | [ ] |
| 103 | ደረጃ ትምህርቲ? | 1. 1. ምንባብንምፅሓፍን ዘይኽእል 2. 2. 8ይ ክፍሊ 3. 3. 9-12 ክፍሊ 4. 4. ኮሌጀ /ዩኒቨርስቲ ዝወደአ/ 5. 5. 2ይ ድግሪን ልዕሊኡን |  | [ ] |
| 104 | እንታይ ብሄረሰብ ኢኹም/ኽን? | 1. 1. ትግራይ 2. 2. ካሊእ፣ ይገለፅ__________ |  | [ ] |
| 105 | ሃይማኖትኩም/ክን እንታይ እዩ? | 1. ኦርቶዶክስ 2. ሙስሊም 3. ፕሮቴስታንት 4. ካቶሊክ 5. ካሊእ፣ይገለፅ__________ |  | [ ] |
| 106 | ናይ ሓዳር ኩነታት ዝምልከት? | 1. ዘይተመርዐወ/ት 2. በዓል/ቲ ሓዳር 3. ዝተፋትሐ/ት 4. ሰብኣያ ዝሞታ/ ሰበይቱ ዝሞተቶ |  | [ ] |
| 107 | ዓይነት ስራሕ? | 1. ናይ መንግስቲ ሰራሕተኛ  2.መንግስታዊ ዘይኮነ ትካል ሰራሕተኛ  3. ነጋዳይ  4. ተምሃራይ  5. መዓልታዊ ሰራሕተኛ  6. ካልእ፣ ይገለፅ________ |  | [ ] |
| 108 | ኣብዚ ገዛኹም ንባዕሎም/ለን ሓዊሱ ክንደይ ልዕሊ 18 ዓመት ሰባት አለኹም | በዘሒ ሰባት___________ |  | [ ] |
| 109 | ወርሓዊ ማእከላይ እቶት ናይ ቤተሰብኩም | ብር___________ |  | [ ] |

**ለ. ባህርያዊ ኩነታት ዝምልከት ሕቶታት**

| ተ.ቁ | ሕቶታት | መልሲ | ናብ ዝቕፅል ሕለፉ | መፍለጢ ቁፅሪ |
| --- | --- | --- | --- | --- |
| **ትንባኾ ምጥቃም ዝምልከት** | | | | |
| 110 | ዝኾነ ይኹን ዓይነት ትንባኾ ከም ሽጋራ ዝመሳሰሉ ተጠቒምኩም /ክን ዶ ትፈልጡ/ጣ | 1. እወ 2. ኣይፋሉን | መልሱ ኣይፋሉን እንተኾይኑ ናብ ሕቶ 116 ሕለፉ | [ ] |
| 111 | ኣብዚ ሐዚ እዋን ዝኾነ ይኹን ትንባኾ ከም ሽጋራ ዝመሳሰሉ የትክኹ ድዮም/ድየን? | 1. እወ 2. ኣይፋሉን | መልሱ ኣይፋሉን እንተኾይኑ ናብ ሕቶ 115 ሕለፉ | [ ] |
| 112 | በቢ መዓልቱ ሽጋራ ትጥቀሙ ዲኹም/ኽን? | 1. እወ 2. ኣይፋሉን |  | [ ] |
| 113 | ተሰታውሰዎ እንተኾይንኩም ንኽንደይ ዓመት ሽጋራ ተጠቒምኩም/ክን? | ______(ዓመት)  ኣየስታውሶን (77) |  | [ ] |
| 114 | ብማእኸላይ ኣብ ሕድሕድ መዓልቲ ክንደይ ሽጋራ ተትክኹ/ኻ? | ___(በዝሒ ሽጋራ) |  | [ ] |
| 115 | መዓልታዊ ሽጋራ ምትካኽ ካብ ዘቋርፁ/ፃ ክንደይ ይኸውን ?  (ኣብ ሓደ ቦታ ጥራሕ ይመላእ) | _____(ብዓመት)  _____(ብኣዋርሕ)  _____(ብሰሙናት)  አየስታውሶን (77) |  | [ ] |
| **ኣልኮሆል ዘለዎ መስተ ዝምልከት** | | | | |
| 116 | ኣብ ሙሉእ ዕድመኦም/አን ኣልኮል ዘለዎ መስተ ሰትዮም/የን ዶ ይፈልጡ/ጣ (ንኣብነት ከም ቢራ ፣ ዋይን፣ ደረቕ ናይ መስተ ዓይነታት? | 1. እወ  2. ኣይፋሉን | መልሱ ኣይፋሉን እንተኾይኑ ናብ ሕቶ 122 ሕለፍ | [ ] |
| 117 | ኣበዚ ዓመት ኣልኮል ዘለዎ መስተ ሰትዮም/የን ዶ ይፈልጡ/ጣ? | 1. እወ  2. ኣይፋሉን |  | [ ] |
| 118 | ኣብዚ ሐዚ እዋን ዝኾነ ዓይነት ኣልኮል ዘለዎ መስተ ትሰትዩ/ያ ዶ? | 1. እወ  2. ኣይፋሉን | መልሱ ኣይፋሉን እንተኾይኑ ናብ ሕቶ 122 ሕለፍ | [ ] |
| 119 | ኣብ ዝሓለፈ 12 ኣዋርሕ ክንደየናይ ኣልኮላዊ መስተ ሰትየ ይብሉ/ላ | 1. መዓልታዊ  2. 5-6መዓልቲኣብ ሰሙን  3. 1-4መዓልቲኣብ ሰሙን  4. 1-3መዓልቲ ኣብ ሰሙን  5. ትሕቲ ሓደ ግዜ ብወርሒ |  | [ ] |
| 120 | ኣብ ዝሓለፈ30 መዓልቲታት ብሓደ ግዜ ክንደይ ዝኣክል ኣልኮላዊ መስተሰትየ ይብሉ/ላ | ________(በዝሒ)  ኣይፈልጦን (77) |  | [ ] |
| 121 | ብማእኸላይ ክንደይ መጠን መስተ ብሰሙን ትሰትዩ/ያ? | ______(በዝሒ መስተ)  ኣይፈልጦን (77) |  | [ ] |
| **ኣመጋግባ ዝምልከት** | | | | |
| 122 | ኣትክልትን ፍራምረን ትምገቡ ዶ ? | 1. እወ 2. ኣይፋሉን | መልሱኣይፋሉን እንተኾይኑ ናብ 125 ሕለፍ | [ ] |
| 123 | ብማእኸላይ ክንደይ መዓልቲ ኣብ ሰሙን ኣትክልትን ፍራምረን ትምገቡ? | ______(በዝሒ መዓልቲ) |  | [ ] |
| 124 | ኣትክልትን ፍራምረን ኣብ እትምገብሉ መዓልቲ ክንደይ ግዜ ብመዓልቲ ትምገቡ? | _______(በዝሒ ማኣዲ ዓይነት ኣትክልትን ፍራምረን) |  | [ ] |
| 125 | ኣሕምልቲ ትምገቡ ዶ ? | 1. እወ 2. ኣይፋሉን | መልሱኣይፋሉን እንተኾይኑ ናብ 129 ሕለፍ | [ ] |
| 126 | ብማእኸላይ ክንደይ መዓልቲ ኣብ ሰሙን ኣሕምልቲ ትምገቡ? | ______(በዝሒ መዓልቲ) |  | [ ] |
| 127 | ኣሕምልቲ ኣብ እትምገብሉ ክንደይ ግዜ ብመዓልቲ ትምገቡ? | ______(በዝሒ ማኣዲ ዓይነት ኣሕምልቲ) |  | [ ] |
| 128 | ምግቢ ኣብ ዝዳለወሉ እዋን ኣብ ገዛኹም ኣየናይ ዓይነት ዘይቲ ወይ ቅብኣት ኢኹም መብዛሕትኡ ግዜ እትጥቀሙ?  (ሓደ ምረፅ) | 1. ካብ ኣትክልቲ ዝተሰርሐ ዘይቲ  2. ጠስሚ  3. ማርጋኒን ዘይቲ  4. ምንም ኣይንጥቀምን  5. ኣይፈልጥን (77) |  | [ ] |
| **ኣካላዊ ምንቅስቃስ ዝምልከት** | | | | |
| 129 | ኣብ ስራሕኹም ዝለዓለ ጉልበት ዝሓቱ ንጥፈታት ከም ተሎተሎ ክተተንፍስ ወይ ድማ ናይ ልቢ ውቅዒት ዘወስኹ ንጥፈታት እንተነኣሰ ን 10 ደቒቓ ዝኣክል ብቐፃልነት ትሰርሑ ዶ? | 1. እወ  2. ኣይፋሉን | መልሱ ኣይፋሉን እንተኾይኑ ናብ ሕቶ 132 ሕለፍ | [ ] |
| 130 | ብማእኸላይ ክንደይ መዓልቲ ኣብ ሰሙን ዝለዓለ ጉልበት ዝሐቱ ንጥፈታት ኣብ ስራሕኹም/ኽን ትሰርሑ/ሓ ? | ________(በዝሒ መዓልቲ) |  | [ ] |
| 131 | ክንደይ ግዜ ብመዓልቲ ዝለዓለ ጉልበት ዝሐቱ ንጥፈታት ኣብ ስራሕኹም/ኽን ትሰርሑ/ሓ? | _____(በሰዓታት)  _____(በደቒቓ) |  | [ ] |
| 132 | እትሰርሕዎ/ዮ ስራሕ ማእኸላይ ጉልበት ዘድልዮም ንጥፈታት ተሎ ተሎ ኣብ ምትንፋስ ወይ ናይ ልቢ ውቅዒት ብማአኸላይ ደረጃ ክውስኽ ዝሕግዙ ከም ፍጥነት ዘለዎ ናይ እግሪ ጉዕዞ ብቐፃልነት እንተነኣሰ ን10 ደቒቓ ዝኣክል ዶ ትንቀሳቐሱ? | 1. እወ 2. ኣየፋሉን | መልሱ ኣይፋሉን እንተኾይኑ ናብ 135 ሕለፍ | [ ] |
| 133 | ብማእኸላይ ኣብ ሰሙን ክንደይ መዓልቲ ማእኸላይ ጉልበት ዘድልዮ ንጥፈት መዓልታዊ ክፋል ስራሕ ትሰርሑ/ሓ? | ________(በዝሒ መዓልቲ) |  | [ ] |
| 134 | ክንደይ ዝኣክል ግዜ ብመዓልቲ ማእኸላይ ጉልበት/ሓይሊ ዘለዎ ስራሕ ብምስራሕ ተሕልፎ/ዮ? | _______(በሰዓት)  _______(በደቒቓ) |  | [ ] |

**ሐ. ጥዕናዊ ኩነታት ዝምከቱ ሕቶታት**

| ተ.ቁ | ሕቶታት | መልሲ | ናብ ዝቕፅል ሕለፉ | መፍለጢ ቁፅሪ |
| --- | --- | --- | --- | --- |
| 135 | ፀቕጢ ደምኩም ንምፍላጥ ብጥዕና በዓል ሞያ ተለኪዕኹም ዶ ትፈልጡ? | 1. እወ 2. ኣይፋሉን | መልሱ ኣይፋሉን እንተኾይኑ ናብ 139 ሕለፍ | [ ] |
| 136 | ዝኾነ በዓል ሞያ ጥዕና በዝሒ ደም ከም ዘለኩም ሓቢሩልኩም ነይሩ ድዩ? | 1. እወ 2. ኣይፋሉን |  | [ ] |
| 137 | ኣብዚ ዓመት በዝሒ ደም ከም ዘለኩም ተሓቢሩልኩም ነይሩ ድዩ? | 1. እወ 2. ኣይፋሉን |  | [ ] |
| 138 | ኣብዚ 2 ሰሙን ናይ ፀቅጢ ደም መድሓኒት ወሲዶም/ን ዶ ነይሮም/ን? | 1. እወ 2. ኣይፋሉን |  | [ ] |
| 139 | ኣብ ቤተሰብኩም ሕማም ፀቅጢ ደም ዘለዎ ዝኾነ ሰብ ኣሎ ድዩ ? | 1. እወ 2. ኣይፋሉን | መልሱ ኣይፋሉን እንተኾይኑ ናብ 141 ሕለፍ | [ ] |
| 140 | ካብ ቤተሰብኩም መን ሕማም ሕማም ፀቅጢ ደም ኣለዎ? | 1. ወለደይ 2. ሓፍተይ 3. ሓወይ |  | [ ] |

**መ. ኣፍልጦ ኩነታት ፀቕጢ ደም ዝምልከቱ ሕቶታት**

| ተ.ቁ | ሕቶታት | መልሲ | ናብ ዝቕፅል ሕለፉ | መፍለጢ ቁፅሪ |
| --- | --- | --- | --- | --- |
| 141 | ብዛዕባ ፀቕጢ ደም ሰሚዕኹም ዶ ትፈልጡ? | 1. እወ  2. ኣይፋሉን | መልሱ ኣይፋሉን እንተኾይኑ ናብ 146 ሕለፍ | [ ] |
| 142 | ፀቕጢ ደም ብምንታይ ይለዓል ? | 1. ናይ ሰውነትና ደም መጠን ምውሳክ 2. ካብ ወለዲ ይሓልፍ 3. ካልእ፣ ይገለፅ________ |  | [ ] |
| 143 | ንፀቅጢ ደም ዘቃልዑ ነገራት እንታይ እንታይ እዮም ? | 1. ዝለዓለ ክብደት ምውሳክ 2. ልዑል ቅብኣት ዘለዎም ምግብታት 3. ዝለዓለ ጨው ብምውሳድ 4. ሽጋራ ብምትካክ 5. መስተ ብምስታይ 6. ኣካላዊ ምንቅስቃስ ብዘይምግባር 7. ዕድመ ብምድፋእ 8. ካልእ፣ ይገለፅ________ |  | [ ] |
| 144 | ፀቅጢ ደም ምክልካል ይከኣል ዶ ? | 1. እወ 2. ኣይፋሉን |  | [ ] |
| 145 | ፀቅጢ ደም ምክልካል ይከኣል ዶ? | 1. ኣካላዊ ምንቅስቃስ ብምግባር 2. ኣልኮላዊ መስተ ብዘይምስታይ 3. ሺጋራ ብዘይምጥቃም 4. ብበዝሒ ቅብኣት ዘለዎም ምግብታት ዘይምምጋብ 5. ብበዝሒ ጨው ዘለዎም ምግብታት ዘይምምጋብ 6. ካልእ፣ ይገለፅ________ |  | [ ] |

**ሠ. ፀቕጢ ደም፣ ቁመትን ክብደት ሰውነትን መጠን መለክዒ ቅጥዒ**

| **ተ.ቁ** | ሕቶ | መልሲ | ናብ ዝቕፅል ሕለፉ | መፍለጢ ቁፅሪ |
| --- | --- | --- | --- | --- |
| **146** | ፀቕጢ ደም(2 ገዜ በቢ 5ተ ደቒቓ ኣፈላላይ ይልካዕ | | | |
|  | ቀዳማይ ንባብፀቕጢ ደም | ________ሲስቶሊክ (ሚ.ሜ ሜርኩሪ)  _____ዲያስቶሊክ (ሚ.ሜ ሜርኩሪ) |  | [ ] |
|  | ካልኣ ይንባብ ፀቕጢ ደም | ________ሲስቶሊክ (ሚ.ሜ ሜርኩሪ)  _____ዲያስቶሊክ (ሚ.ሜ ሜርኩሪ) |  | [ ] |
|  | ሳልሳይ ንባብ ፀቕጢ ደም | ________ሲስቶሊክ (ሚ.ሜ ሜርኩሪ)  _____ዲያስቶሊክ (ሚ.ሜ ሜርኩሪ) |  | [ ] |
| 147 | ቁመት | _______(ብሴንትሜትር) |  | [ ] |
| 148 | ክብደት | _________ (ብኪ.ግ) |  |  |
| 149 | መጠን ክብደት ሰውነት ኢንዴክስ (ክብደት/ቁመት^2^) | ________ (ኪ.ግ/ሜ^2^) |  |  |

**ንዝሃብኩምና ግዜ ብጣዕሚ የቐንየለይ!**
